# Evaluation of Bag-Valve-Mask Ventilation in Manikin Studies: What Are the Current Limitations?

**DOI:** 10.1155/2016/4521767

**Published:** 2016-05-16

**Authors:** A. Khoury, F. S. Sall, A. De Luca, A. Pugin, S. Pili-Floury, L. Pazart, G. Capellier

**Affiliations:** ^1^Department of Emergency Medicine & Critical Care, University of Franche-Comté, Medical Centre, 25000 Besançon, France; ^2^Clinical Investigation Centre, INSERM CIC-1431, University of Franche-Comté, Medical Centre, 25000 Besançon, France; ^3^Department of Anaesthesia and Critical Care, University of Franche-Comté, Medical Centre, 25000 Besançon, France; ^4^Monash University, Melbourne, VIC 3800, Australia

## Abstract

*Introduction.* Manikin-based studies for evaluation of ventilation performance show high heterogeneity in the analysis and experimental methods used as we pointed out in previous studies. In this work, we aim to evaluate these potential limitations and propose a new analysis methodology to reliably assess ventilation performance.* Methods.* One hundred forty healthcare providers were selected to ventilate a manikin with two adult self-inflating bags in random order. Ventilation parameters were analysed using different published analysis methods compared to ours.* Results.* Using different methods impacts the evaluation of ventilation efficiency which ranges from 0% to 45.71%. Our new method proved relevant and showed that all professionals tend to cause hyperventilation and revealed a significant relationship between professional category, grip strength of the hand keeping the mask, and ventilation performance (*p* = 0.0049 and *p* = 0.0297, resp.).* Conclusion.* Using adequate analysis methods is crucial to avoid many biases. Extrapolations to humans still have to be taken with caution as many factors impact the evaluation of ventilation performance. Healthcare professionals tend to cause hyperventilation with current devices. We believe this problem could be prevented by implementing monitoring tools in order to give direct feedback to healthcare professionals regarding ventilation efficiency and ventilatory parameter values.

## 1. Introduction

Bag-valve-mask (BVM) ventilation is mainly used in prehospital settings to ventilate patients in respiratory failure and/or cardiac arrest. This difficult procedure has attracted the attention of scientists since the eighties. Indeed, many studies have investigated factors such as ventilation techniques [1–6], ventilation devices [7–12], and operator characteristics and skills [6, 8, 9, 11] that may impact its performance. Most of them were carried out on manikin-based simulators. However, we previously reported that the use of this simulation tool might significantly impair peak pressure, tidal volume, and leakage measurements if the manikin intrinsic compliance and resistance are not properly considered when processing the recorded data. Indeed, we showed, on one of the most commonly used manikins, measurement biases of tidal volume ranging from 23 mL to 62 mL which could be the consequence of important inaccuracies which does not reflect exactly the human airway anatomy [13, 14]. Moreover, in a recent literature review we published, we noticed that scientists use different methodology to evaluate ventilation performance and we pointed out their heterogeneity in terms of both judgment criteria and analysis methods [15]. These methods do not consider ventilation variability within and between persons and do not give a relevant assessment of ventilation efficiency. Their potential impact on the evaluation of manual ventilation performance remains to be proved.

All these factors raise the question of the reliability of manikin-based studies. This research consists of an evaluation of the impact of these factors on manual ventilation performance, in terms of analysis methodology, system-based simulations, or operator characteristics. We also propose a new analysis method, able to provide a chronological view of ventilation variability in order to accurately assess manual ventilation performance in bench conditions.

## 2. Materials and Methods

### 2.1. Protocol Design

We conducted this experimental trial at the Department of Emergency Medicine and Critical Care at the University of Franche-Comté Medical and Trauma Centre, Doubs Fire Department, and Jussieu Ambulance Services. The study protocol was submitted to the French data protection authority (Commission Nationale de l'Informatique et des Libertés, CNIL, registration number 1645179). The need for ethical approval was waived by the institutional ethics committee (Comité de Protection des Personnes CPP Est II). We enrolled healthcare professionals who were still in service, aged over 18 years, coming from different emergency structures (University of Franche-Comté Medical and Trauma Centre, Doubs Fire Department, and Jussieu Ambulance Services). Data from 140 volunteers were collected.

Prior to start, participants signed informed consent and fulfilled a questionnaire. We determined the size of the hand squeezing the bag and the grip strength of both hands using a tape measure and a Takei® dynamometer, respectively. Participants were instructed to ventilate with a bag-valve-mask a manikin simulating a 75 kg adult patient in respiratory arrest as they are used to do in their everyday practice. They were blinded regarding their ventilation performance. Because participants were not able to see the manikin chest rise, they were asked to ventilate the manikin for one minute before the tests in order to accustom themselves to the bench model. Then, participants manually ventilated the manikin with two adult self-inflating bags (Laerdal Bag® II and Ambu Spur® II with a reservoir volume of 2900 and 2600 mL, resp.) for five minutes each in a random order. Ventilation was performed in a standing position using a standard technique without chest compression, that is, one hand keeping the mask on the manikin's face and the other hand squeezing the bag.

### 2.2. Experimental Bench Model

A Laerdal Airway Management Trainer manikin (Laerdal Medical, Stavanger, Norway) was installed on a stretcher. The manikin's lungs were bypassed and directly connected to an ASL 5000® breathing simulator (IngMar Medical, Ltd., Pittsburgh, PA, USA) with a short respiratory hose (ID = 2.2 cm, *L* = 36 cm). A single compartment lung model was set with compliance of 70 mL·cmH_2_O^−1^ and resistance of 3.5 cmH_2_O·L^−1^·s. The intrinsic resistance and airway dead space of the manikin were evaluated for an accurate definition of our patient model [14]. Thus, the manikin's dynamic airway resistance ranged from 2 to 5 cmH_2_O·L^−1^·s for peak flows ranging from 20 to 120 L·min^−1^. Tidal volumes (*V*_*T*_), lung peak flows (PF_Lung_), and inspiratory and expiratory times (*I*_time_, *E*_time_) were measured inside the ASL 5000 lung simulator and recorded for each ventilation cycle directly by the ASL SW3.3.106 software.

We also used two VTT® flowmeters (Jeulin, MediaScience, Haute-Normandie, France) to measure gastric and BVM insufflation flows.

### 2.3. Data Treatment

VTT sensor signals were triggered using MATLAB® (version R2008b, MathWorks, Natick, MA, USA) in order to detect each ventilation phase of each ventilatory cycle. Gastric tidal volumes (*V*_*G*_) and BVM insufflation volumes (*V*_ins_) were obtained through a time-related integral transformation of the flowmeters signals over the insufflation period of each cycle. Instantaneous ventilation rates (*V*_*R*_) were calculated by measuring the period between each insufflation phase. We recalibrated tidal volume measurements according to peak insufflation flows obtained by the VTT sensor in order to compensate *V*_*T*_ deviation induced by the compliance of the dead space of the manikin [14].

### 2.4. Ventilation Performance Analyses

In a previous literature review, we showed a wide heterogeneity in the definition of successful ventilation, in terms of both analysis methods and judgment criteria [15]. In this paper, we aim to apply, on a unique database, the three main methods described below in order to study their impact on results:

(i) Method 1: an overall mean value analysis, that is, the analysis of the global mean value of each ventilatory parameter for all subjects combined together. This method does not take into account inter- and intravariability of ventilatory parameters.
(ii) Method 2: an individual mean value analysis, that is, the analysis of the mean value of each ventilatory parameter for each single subject. Compared to Method 1, it considers the variability between persons but not within person.
(iii) Method 3: a breath-by-breath analysis of each ventilatory parameter. This method takes into account variability between and within persons but it does not correlate it with time.

Although the main judgment criteria described in our review were tidal volume (*V*_*T*_) and ventilation rate (*V*_*R*_), different tolerance ranges were used to define ventilation efficiency (Table 1).

Finally, considering that none of these approaches is able to provide a clear understanding of ventilation variability [15], we worked out a new analysis approach. A specific algorithm has been designed to observe variations of a whole ventilation sequence. It consists in segmenting every ventilation test into sliding windows of one-minute length each with a shift of three ventilation cycles each time. This will enable us to consider intra- and interindividual and time variability of ventilatory parameters.

This algorithm evaluates the performance of a one-minute window depending on two judgment parameters: tidal volume (*V*_*T*_) and ventilation rate (*V*_*R*_). We previously reported on the lack of consensus regarding adequate *V*_*T*_. However, a recent study by Lyazidi et al. led us to conclude that *V*_*T*_ ranging from 4 to 8 mL·kg^−1^ may be considered adequate, as long as they are measured inside the artificial lung [14, 16]. Thus, these threshold values were applied to our patient model (IBW: 75 kg) to define our tolerance range for adequate *V*_*T*_ from 300 to 600 mL. Similarly, we considered *V*_*R*_ between 8 and 15 bpm to be adequate regarding our patient model (no respiratory pathology). Therefore, we distinguish three situations to assess the performance of a sliding window according to *V*_*T*_ and *V*_*R*_ measurements during the one-minute period:

(i) 1st situation: *V*_*R*_ ≤ 15 bpm, mean *V*_*T*_ ≤ 600 mL, and/or there are at least 8 adequate ventilation cycles (with *V*_*T*_ between 300 and 600 mL). This is considered to be efficient as there is no significant risk of dynamic hyperinflation.
(ii) 2nd situation: *V*_*R*_ ≤ 15 bpm, mean *V*_*T*_ ≤ 600 mL, and/or the number of adequate ventilation cycles <8. This ventilation is considered insufficient as it may lead to poor tissue oxygenation.
(iii) 3rd situation: *V*_*R*_ > 15 bpm and/or mean *V*_*T*_ > 600 mL. This condition underlines global hyperventilation in the last minute period. In this case, where high *V*_*R*_ and *V*_*T*_ may increase intrathoracic pressure leading to pulmonary barotrauma and impairing haemodynamics, ventilation is considered excessive.

**Figure 1:**
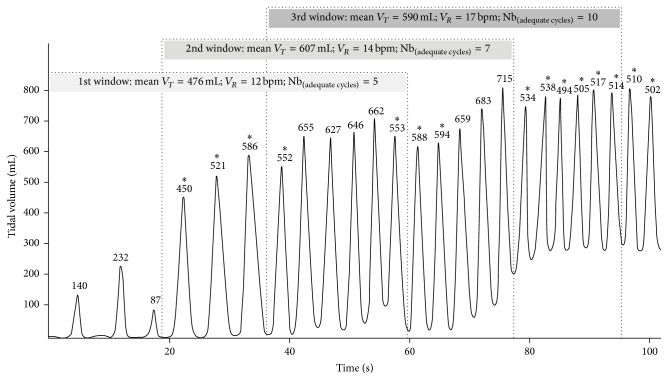
Operating process of the new analysis algorithm. This figure shows the evaluation of one-minute sliding windows with a shift of three ventilation cycles. The 1st window is considered insufficient as there are only 5 adequate ventilation cycles. The 2nd window is excessive as mean *V*_*T*_ > 600 mL. The 3rd window is excessive as *V*_*R*_ > 15 bpm. Global ventilation performance of the whole test is considered excessive as the majority of sliding windows are excessive.  ^*∗*^Adequate ventilation cycles.

In order to explain how to implement this new analysis method, we tried to illustrate its operating process in Figure 1 by taking example on a ventilation curve.

The program appraises the global performance of the five-minute ventilation test by considering every sliding window performance and giving the general trend of the whole ventilation sequence. This novel method will be used to evaluate which factors influence manual ventilation performance of our healthcare professionals.

### 2.5. Statistical Analysis

Continuous data are expressed as mean ± SD. Results are presented as percentages for nominal variables. Chi2 and Fisher exact test were used to compare professional categories and experience, BVM type, hand size, and hand grip strengths between the three different performance levels. Odds ratio, estimated by logistic regression, was used to analyse performance level for the multivariate model. A *p* value lower than 0.05 was considered to be statistically significant. Statistical analysis was performed with SAS 9.3 for Windows (SAS Institute Inc., Cary, NC, USA).

## 3. Results

Forty-five physicians (29 emergency medicine physicians and 16 anaesthesia/critical care physicians), forty-five nurses (27 anaesthesia nurses and 18 emergency medicine nurses), and fifty rescuers (31 firefighters, 17 emergency medical technicians, and 2 Red Cross first-aid rescuers) were enrolled in the study. Their professional experience ranged from less than one year to greater than 20 years. The mean population age was 37 ± 9 years. One-third of the volunteers were women. Fifty-five participants thought that their performance was “good,” 83 thought that it was “medium,” and only one thought that it was “bad.” The detailed characteristics of the population are shown in Table 2.

### 3.1. Evaluation of the Different Analysis Methods and Definitions of Adequate Ventilation

Two hundred eighty ventilation tests have been recorded as each volunteer performed twice the five-minute ventilation test with two different BVM devices. Three different analysis methods and five definitions of ventilation efficiency were applied to this database to evaluate the manual ventilation performance of healthcare professionals. More than 54,000 ventilation cycles have been analysed. Ventilatory parameter values and manual ventilation performance results are reported in Tables 3 and 4.

Using the overall mean value analysis (Method 1), tidal volume was 333.94 ± 124.19 mL and ventilation rate was 24 ± 9 bpm with 25% of the recorded *V*_*R*_ above 29 bpm (Table 3).

Whatever the definition used, mean values of *V*_*T*_ and *V*_*R*_ obtained with this method are not within the tolerance range, hence 0% of adequate ventilation (Table 4). Every ventilatory parameter seems to have high dispersion with a SD representing more than 30% of the mean value. Therefore, we evaluated ventilation variability within and between persons. Intraindividual variability, from one ventilation cycle to another, accounts on average for 15.6% and 15.4% of the mean *V*_*T*_ and *V*_*R*_, respectively. In some participants, it reaches 86.1% and 66.0%. Standard deviation between the mean values of each participant is 132 mL for the mean *V*_*T*_ and 8 bpm for the mean *V*_*R*_. This interindividual variability represents approximately 38% of the mean value of each parameter. Indeed, the maximum mean *V*_*T*_ and *V*_*R*_ are 877 mL and 53 bpm and the minimum ones are 47 mL and 8 bpm. Mean BVM insufflation volume (*V*_ins_) was 590.20 ± 193.31 mL while mean *V*_*T*_ was 333.94 ± 124.19 mL, hence a leakage volume of approximately 200 mL (37% of *V*_ins_). Leaks are also visible when comparing mean lung peak flow (39.99 ± 16.53 L·min^−1^) with mean BVM peak flow (69.26 ± 28.07 L·min^−1^).

Unlike Method 1, the influence of Methods 2 and 3 (the individual mean value analysis and the breath-by-breath analysis) on ventilation performance depends on the definition of adequate ventilation (Table 4). Indeed, for example, ventilation efficiency ranges from 0% to 45.71% and from 0.41% to 40.01% for Methods 2 and 3, respectively. Different definitions of efficient ventilation have thus an impact on results according to the analysis method used. Similarly, the analysis method can significantly impact performance results depending on the definition. For example, for Methods 2 and 3, Definition 4 gives, respectively, 25.71% and 14.35% of adequate ventilation (*p* < 0.001).

### 3.2. Ventilation Performance Results Using Different BVM Models

The results show that the use of different BVM models (Laerdal Bag II and Ambu Spur II) does not significantly affect manual ventilation performance (*p* = 0.79). Among 280 ventilation tests realized, 121 and 122 were inadequate for Ambu and Laerdal bags, respectively.

### 3.3. Ventilation Performance and Human Factors Using a New Analysis Method

Our new method showed only 21 (7.50%) efficient ventilation tests while 37 (13.21%) were insufficient and 222 (79.29%) were excessive.

**Figure 2:**
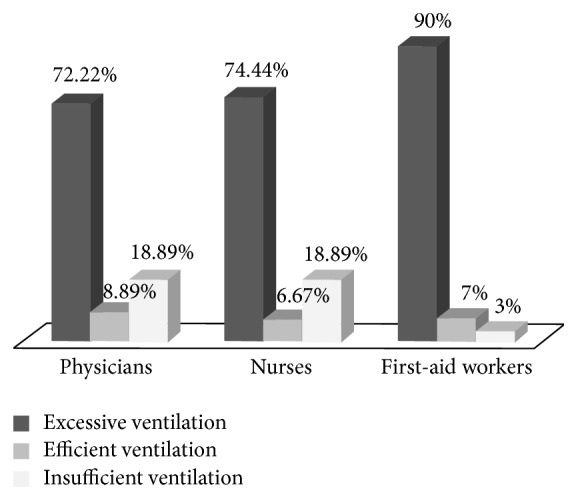
Percentage of excessive, efficient, and insufficient ventilation tests for professional categories (*n* = 280).

Moreover, statistical analyses have revealed no effect of hand size (*p* = 0.31), professional experience (*p* = 0.48), and grip strength of the hand squeezing the bag (*p* = 0.15). However, a significant relationship between participants' professional category and ventilation performance has been shown (Figure 2, *p* = 0.0049). While there is no statistical difference regarding the percentage of efficient ventilation, the proportion of excessive ventilation was significantly higher for rescuers (90%) than for nurses (74.44%) and physicians (72.22%) who consequently had higher percentages of insufficient ventilation (18.89% versus 3%).

**Figure 3:**
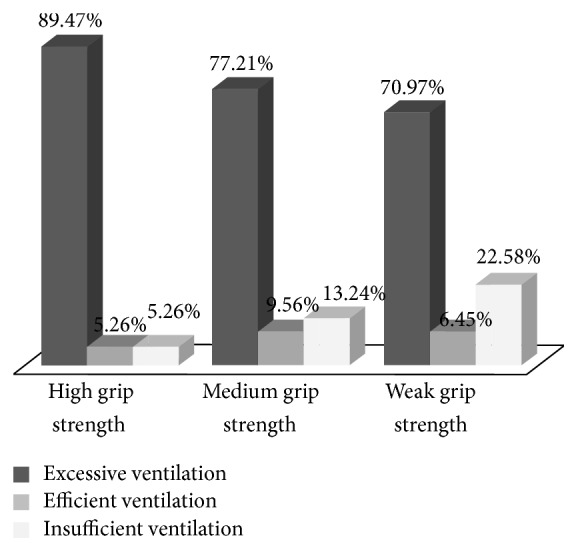
Percentage of excessive, efficient, and insufficient ventilation tests for grip strength categories of the hand keeping the mask (*n* = 274^*∗*^, ^*∗*^6 missing values).

Univariate analysis has revealed that the grip strength of the hand keeping the mask has an impact on ventilation performance (Figure 3, *p* = 0.0297). Indeed, excessive ventilation proportions are increasing with grip strength of the hand keeping the mask (70.97%, 77.21%, and 89.47% for weak, medium, and high grip strength, resp.) while insufficient ones are decreasing (22.58%, 13.24%, and 5.26%, resp.). Furthermore, ventilation efficiency is higher for participants with medium grip strength (9.56%) than for those with a high one (5.26%) or a weak one (6.45%). However, multivariate analysis shows that grip strength has less influence than professional category and only this last stays significant (*p* = 0.0286).

## 4. Discussion

### 4.1. Factors Related to Analysis Methodology

In a previous manuscript, we identified three different analysis methods and five definitions of adequate ventilation used in several reviewed studies [15]. We have pointed out the heterogeneity of these approaches and their inability to consider ventilatory parameter variability within and between persons. We suggested that this could have a significant impact on results. Thus, this study was an opportunity to verify this hypothesis in a single database and to quantify its real impact on manual ventilation performance. Our findings showed that analysis methodology is the factor that has the most important effect on ventilation performance as it varies from 0% to 45.71% according to different analysis methods and definitions of ventilation efficiency. This would be due to the relative variability of *V*_*T*_ and *V*_*R*_ within persons which represents approximately 15% of the mean value of each and reached up to 86% and 66%, respectively, in some participants. Variability between persons was even more significant (about 38% of the mean value of each parameter) as mean *V*_*T*_ and *V*_*R*_ ranged from 47 to 877 mL and from 8 to 53 bpm.

For these reasons, we defined a new analysis method that allows a chronological evaluation of ventilatory parameters by segmenting a whole ventilation test into one-minute sliding windows. Figure 1 clearly shows the significance of ventilation variability and its impact on the evaluation of ventilation performance. In this example, our algorithm is able to detect a first period of insufficient ventilation with low tidal volumes (1st window) followed by a longer period of hyperventilation with excessive tidal volumes and ventilation rates (2nd and 3rd windows). This analysis method enables us to make a reliable assessment of the performance of the whole ventilation test. In this case, ventilation is considered excessive as there are a majority of excessive ventilation windows. However, if we had used one of the existing analysis methods that do not allow studying ventilation variability, this ventilation test would have been considered adequate. Indeed, Methods 1 and 2 would have found a mean *V*_*T*_ of 523 mL and a mean *V*_*R*_ of 14 bpm, which are both into their target range, and Method 3 would have resulted in 58% (14/24) of efficient ventilation cycles.

With our novel method, 280 ventilation tests realized by 140 healthcare professionals were analysed and we found that only 21 (7.50%) were efficient; the remaining 259 tests (92.50%) can be considered potentially deleterious. However, using the overall mean value analysis method, the BVM ventilation is ineffective with 0% of efficient ventilation tests according to different definitions reviewed.

Among the 92.50% of inadequate ventilation tests, 79.29% were excessive which may impair haemodynamics and induce pulmonary barotrauma, and 13.21% of them were insufficient which may cause hypoxia despite the fact that most of the participants thought their ventilation was adequate. This confirms on a larger sample size the results reported in previous studies [11, 17–20].

### 4.2. Factors Related to Manikin and BVM Models

Manikin-based simulations could influence manual ventilation performance as the anatomical design of facemasks is not particularly adapted to the manikin's face shape [21]. Indeed, we found major differences between BVM insufflation volume and *V*_*T*_, leading to a mean leak volume of 219 mL which represents ≈37% of the mean insufflation volume. This could be explained by the difficulty in keeping a real airtight seal [21]. Even if we avoided biases related to manikin compliance and resistance by recalibrating data, manikins used in ventilation studies cannot reflect exactly human respiratory mechanics. Another problem is the absence of chest movements which could disrupt healthcare professionals and their appreciation of ventilation quality provided. However, asking them to train on the bench model before tests may have minimized these biases. Although clinical trials and animal studies enable evaluating accurately ventilation efficiency by measuring physiological variables such as arterial blood gases, manikin-based studies remain the most widely used models as they are easy to implement with less stringent ethical rules and provide also the capability for standardization and isolation of key characteristics [13–15].

Furthermore, our study showed that the use of different BVM models has no impact on ventilation performance, although many healthcare professionals perform ventilation more with Ambu bags than with Laerdal ones (115 versus 42). A similar result was obtained by Augustine et al. who demonstrated that there was no obvious relationship between various BVM models used and the average tidal volume delivered [9]. We think that future development of new technologies may ensure a better control of manual ventilation efficiency.

### 4.3. Factors Related to Human Characteristics and Skills

In order to determine which human factors can affect manual ventilation performance, operator characteristics such as professional categories and experience, hand grip strength, and hand size have been evaluated.

There was no influence of professional experience and hand size on manual ventilation performance. These results were similar to those obtained by Otten et al. [4]. The fact that professional experience does not impact ventilation performance means that ventilation self-improvement is difficult to achieve with current devices. However, multivariate analysis has shown that the most influencing factor was professional category. While all healthcare workers tend to hyperventilate the manikin, physicians and nurses have lower rate of excessive ventilation. This may be related to higher expertise in physiology and respiratory care. This trend to perform hyperventilation has been shown in the literature [17, 19, 20]. High ventilation rates could also be due to the rapid refilling of the bag which can induce a reflex to squeeze and ventilate the manikin as soon as the bag reinflates [22, 23]. Hyperventilation is also accentuated by high grip strength of the hand keeping the mask. This can be explained by the greater ability to avoid leaks with high grip strength resulting in higher tidal volumes provided to the manikin. Keeping the mask on the manikin's face with one hand is a difficult exercise requiring a strong effort, which may possibly cause hand muscles tetany and induce poor mask-to-face airtightness. Therefore, professionals having high grip strength are more likely to hyperventilate patients than those with a weak one, for whom excessive insufflation volumes are compensated by large amounts of leaks [4]. Some studies have theorized that the difference in tidal volume is related to the ability to obtain a good mask-to-face seal [7, 9]. Conversely, Lee et al. and Augustine et al. showed that physical aspects including hand size, volume, and grip strength had no correlation with tidal volume [6, 9].

### 4.4. Limitations

We proposed a novel analysis method that is more relevant as it enables scientists to observe variability within and between persons, but it still has some limitations. Actually, only the correlation with clinical data, which remain the only real indicators of ventilation performance, will help to validate this new method. Therefore, we still cannot confirm that bag-valve-mask ventilation performance described in this study is a reliable representation of what could be obtained in clinical trials. Moreover, we only conducted this study in three different emergency structures. Even if a low ventilation performance rate has also been reported by other studies, diverse findings could be obtained in other centres where Basic Life Support training courses could differ.

## 5. Conclusion

Many factors may have an impact on manual ventilation performance. In order to accurately assess human factors, it is important to use adequate analysis methods to avoid biases related to methodology. The important variability within and between persons proves the relevance of our new analysis method, which allows observing ventilatory parameter variability on an entire ventilation period. We showed, with this novel method, that professional category and grip strength of the hand keeping the mask have a significant impact on manual ventilation performance. This study confirms that the evaluation of bag-mask ventilation performance is complex and cannot be fully determined on a manikin model. Extrapolations to humans have to be taken with caution. However, we can argue that healthcare professionals perform hyperventilation in most cases and have difficulties in performing adequate manual ventilation with current devices. We believe this problem could be prevented by implementing monitoring tools in order to give direct feedback to healthcare professionals regarding ventilation efficiency and ventilatory parameter values.

## Figures and Tables

**Table 1:** Different definitions and judgment criteria identified in the literature and ILCOR guidelines.

Judgment criteria	Definition 1	Definition 2	Definition 3	Definition 4	Definition 5
Tidal volume	450–525 mL^*∗*^	400–600 mL	—	—	450–525 mL
Ventilation rate	—	—	8–10 bpm^*∗∗*^	10–15 bpm	8–10 bpm

^*∗*^6-7 mL·kg^−1^, 75 kg of IBW; ^*∗∗*^bpm: breaths per minute.

**Table 2:** Characteristics of study population (*n* = 140).

*Mean age ± SD (years)*	37.28 ± 8.97
*Sex (n, %)*	
Female	47 (33.57%)
Male	93 (66.43%)
*Professional category (n, %)*	
Physicians	45 (32.14%)
Nurses	45 (32.14%)
First-aid workers	50 (35.71%)
*Professional experience (n, %) *	
High (≥10 years)	63 (45.00%)
Medium (5 ≤ *n* < 10 years)	36 (25.70%)
Little (<5 years)	41 (29.30%)
*Handedness (n, %)*	
Right-handed	118 (84.29%)
Left-handed	14 (10.00%)
Ambidextrous	8 (5.71%)
*Size of the hand squeezing the bag (n, %)*	
Large (≥23 cm)	21 (15.00%)
Medium (19 ≤ *n* < 23 cm)	101 (72.14%)
Small (15 ≤ *n* < 19 cm)	18 (12.86%)
*Grip strength (n, %)*	
Hand squeezing the bag	
High (≥40 kgF)	53 (37.90%)
Medium (20 ≤ *n* < 40 kgF)	65 (46.40%)
Weak (0 ≤ *n* < 20 kgF)	22 (15.70%)
Hand keeping the mask^*∗*^	
High (≥40 kgF)	38 (27.70%)
Medium (20 ≤ *n* < 40 kgF)	68 (49.60%)
Weak (0 ≤ *n* < 20 kgF)	31 (22.60%)
*BVM type used frequently (n, %)*	
Ambu®	76 (54.29%)
Laerdal®	3 (2.14%)
Both	39 (27.86%)
Neither	22 (15.71%)
*Estimated manual ventilation performance (n, %)* ^**∗∗**^	
Good	55 (39.57%)
Medium	83 (59.71%)
Bad	1 (0.72%)

SD: standard deviation; ^*∗*^3 missing values; ^*∗∗*^1 missing value.

**Table 3:** Ventilation parameter values measured during all the 5-minute ventilation tests (*n* = 280), realized with 140 participants ventilating with two different BVM.

Variable	Mean (SD)	Lower quartile	Upper quartile
Instantaneous ventilation rate (*V*_*R*_, bpm)	24.09 (9.47)	17.20	29.09
Tidal volume (*V*_*T*_, mL)	333.94 (124.19)	245.60	419.95
BVM insufflation volume (*V*_ins_, mL)	590.20 (193.31)	458.11	723.40
Gastric tidal volume (*V*_*G*_, mL)	37.58 (25.13)	18.92	52.43
Lung peak flow (PF_Lung_, L·min^−1^)	39.99 (16.53)	28.40	50.16
BVM peak flow (PF_BVM_, L·min^−1^)	69.26 (28.07)	49.16	85.92
Gastric peak flow (PF_*G*_, L·min^−1^)	5.35 (4.33)	2.53	7.34

**Table 4:** Manual ventilation efficiency (*n* (%)) using different analysis methods and definitions. *n* = 140 healthcare professionals for Methods 1 and 2; *n* = 54,770 ventilation cycles for Method 3.

Analysis methods	Definition 1	Definition 2	Definition 3	Definition 4	Definition 5
Method 1 (overall mean value analysis)	0 (0.00%)	0 (0.00%)	0 (0.00%)	0 (0.00%)	0 (0.00%)
Method 2 (individual mean value analysis)	27 (19.29%)	64 (45.71%)	0 (0.00%)	36 (25.71%)	0 (0.00%)
Method 3 (breath-by-breath analysis)	9232 (16.86%)	21913 (40.01%)	1883 (3.44%)	7860 (14.35%)	222 (0.41%)
